# Association between blood cadmium levels and malnutrition in peritoneal dialysis

**DOI:** 10.1186/1471-2369-15-17

**Published:** 2014-01-16

**Authors:** Ching-Wei Hsu, Ja-Liang Lin, Dan-Tzu Lin-Tan, Wen-Hung Huang, Kuan-Hsing Chen, Tzung-Hai Yen

**Affiliations:** 1 Department of Nephrology and Division of Clinical Toxicology, Chang Gung Memorial Hospital, 199, Tung-Hwa North Road, Taipei, Taiwan; 2 Department of Nephrology and Division of Clinical Toxicology, Lin-Kou Medical Center, Taoyuan, Taiwan; 3 Chang Gung University and School of Medicine, Taipei, Taiwan

**Keywords:** Blood cadmium levels, Chronic peritoneal dialysis, Inflammation, Malnutrition

## Abstract

**Background:**

Malnutrition is associated with an increased risk of cardiovascular death and may cause protein-energy wasting in individuals with chronic kidney disease. A previous study demonstrated that blood cadmium levels (BCLs) were associated with malnutrition in maintenance hemodialysis (MHD) patients. However, the correlation between cadmium exposure and malnutrition remains unclear in chronic peritoneal dialysis (CPD) patients. This study examined the possible adverse effects of environmental cadmium exposure in CPD patients.

**Methods:**

A total of 301 CPD patients were enrolled and divided into 3 study groups based on the following BCL tertiles: low (<0.19 μg/L), middle (0.19–0.39 μg/L), and high (>0.39 μg/L). Demographic, hematological, biochemical, and dialysis-related data were obtained for analysis. The analysis also included values of nutritional and inflammatory markers.

**Results:**

The BCLs of CPD patients were lower than those of MHD patients. At baseline, patients in the high BCL group were older and had a higher prevalence of diabetes mellitus but lower serum albumin, creatinine, and phosphate levels than the patients in the other 2 groups. After adjusting for potential variables, stepwise backward multiple linear regression analysis revealed that age and alanine aminotransferase levels were positively associated with logarithmic transformation of BCLs (log BCLs), while serum albumin levels were negatively associated with log BCLs in CPD patients. The log BCLs were a significant determinant (beta coefficient ± standard error = -0.185 ± 0.074; *P* = 0.013) of nutritional status and significantly associated with the presence of malnutrition (odds ratio = 2.64; 95% confidence interval: 1.07–6.48; *P* = 0.035) in CPD patients after adjustment for related variables.

**Conclusions:**

BCL is significantly associated with nutritional status and malnutrition in CPD patients. Therefore, it is important for CPD patients to avoid environmental exposure to cadmium such as through smoking and consumption of cadmium-rich foods.

## Background

Cadmium is widely known as a toxic metal [1-3]. Environmental and occupational exposure to cadmium is associated with several diseases, including renal dysfunction [2], bone disease [2] and cancers [3]. Occupational studies have shown that cadmium exposure is associated with increased all-cause and cancer-related mortality [2]. Population-based studies conducted in heavily polluted areas have also linked cadmium exposure to an increased risk of cancer-related, cardiovascular-related, and all-cause mortality [4,5]. Recently, the US national survey demonstrated a direct correlation between environmental cadmium exposure and all-cause, cancer, and cardiovascular disease mortality among adults [6].

Cadmium has been shown to accumulate in the bones of patients with end-stage renal disease (ESRD), and increased blood cadmium levels (BCLs) have been observed in maintenance hemodialysis (MHD) patients [7-9]. Additionally, our recent cross-sectional study demonstrated that BCLs are associated with malnutrition and inflammation in MHD patients [10]. Furthermore, an 18-month longitudinal study [11] demonstrated that BCLs are related to all-cause mortality in diabetic MHD patients. However, the clinical significance of BCLs in chronic peritoneal dialysis (CPD) patients remains unknown, and the possible adverse effects of environmental cadmium exposure in CPD patients were not identified.

Recent evidence from clinical studies [12-15] indicated that protein-energy wasting and residual renal function are associated with short-term mortality in ESRD patients. Correcting these risk factors offers a potential means for reducing the mortality rates of CPD patients [15,16]. Therefore, it is important to identify correctable factors associated with residual renal function, inflammation, and/or malnutrition for the welfare of these patients. However, the correlations between BCLs and residual renal function, inflammation, and malnutrition in CPD patients remain unclear.

Therefore, we conducted a cross-sectional study to evaluate the relationship between BCLs and health status by performing clinical examinations of CPD patients.

## Methods

### Ethics statement

This clinical study was conducted in accordance with the Declaration of Helsinki and was approved by the Medical Ethics Committee of Chang Gung Memorial Hospital (CGMH), a tertiary referral medical center in Taiwan. All patients provided written informed consent for the collection of data and subsequent analysis and the approval from Institutional Review Board was obtained. All individual information was securely protected (by delinking identifying information from main data set) and available to investigators only. Furthermore, all the data were analyzed anonymously.

### Patients

All study participants were recruited from the Peritoneal Dialysis Center of Lin-Kou Medical Center, CGMH. Only patients undergoing CPD for more than 6 months were enrolled. Subjects with a history of occupational exposure to heavy metals or metal intoxication were excluded, as well as those living in metal-contaminated areas or near the cadmium-using factories, those with malignancies, and those under 18 years of age. A total of 301 patients were included. The CPD prescription for each patient was based on the peritoneal membrane characteristics, which were determined by peritoneal equilibration tests. Intermittent therapies were used primarily for patients with high transport characteristics, and continuous therapies were used for those with average or low transport characteristics. Of these patients, 51 were treated with automated PD and 250 underwent continuous ambulatory PD, including 1 patient with 3 exchanges per day, 164 patients with 4 exchanges per day (57 with 1.5 L/exchange, 101 with 2 L/exchange, and 6 with 2.5 L/exchange), 22 patients with five 1.5-L exchanges, and 63 patients with five 2-L exchanges per day. Low-calcium (1.5 or 1.25 mmol/L) or standard-calcium dialysate containing glucose (Na^+^, 135 mmol/L; lactate, 35 mmol/L; Ca^2+^, 1.75 mmol/L) and icodextrin-based (7.5 g/dL) dialysate were prescribed for each patient according to the peritoneal transport characteristics and serum calcium levels required to maintain adequate ultrafiltration and normal calcium levels. The dialysis prescription was aimed at obtaining a total Kt/V of at least 1.8 per week.

The presence of primary renal disease and comorbid conditions was determined after an in-depth review of the medical records, including the history and physical examination, progress notes, discharge summaries, consultations, and biopsy results. Patients with diabetes mellitus (DM) were defined as those with a history of diabetes diagnosed by a physician or any 2 subsequent fasting glucose levels greater than 126 mg/dL. The presence of cardiovascular disease (CVD), including cerebrovascular disease, coronary artery disease, congestive heart failure, and peripheral vascular disease, was recorded. History of smoking, alcohol consumption, and drug use were also recorded.

In order to assess the BCLs of the general population in Taiwan, we also enrolled 63 healthy volunteers who had normal renal function and no history of heavy metal contamination or intoxication.

### Measurement of BCLs

To ensure that the blood samples of the CPD patients were not contaminated with cadmium during tests, the samples were collected in cadmium-free plastic tubes. At least two blood samples were obtained from each patient before and 4 h after peritoneal dialysis. BCLs were measured using a previously described method [17]. Briefly, 900 μL of a modifier solution of NH_4_H_2_PO_4_ + HNO_3_ + Triton X-100 in deionized water and 100 μL of whole blood or 100 μL of modifier solution and 900 μL dialysate were added to a 1.5-mL Eppendorf tube and immediately shaken. After overnight storage in a refrigerator, the tubes were warmed to room temperature and then whirl-mixed for 5–10 s. The diluted blood sample was transferred to graphite furnace sampler cups. The cadmium levels of the acid-digested samples were measured by electrothermal atomic absorption spectrometry (SpectrAA-200Z; Varian, Palo Alto, CA, USA) with Zeeman’s background correction and an L’vov platform. Both internal and external quality control procedures were used. A certified commercially prepared product (Seronorm Trace Elements; Sero AS, Billingstads, Norway) was used to determine the intra-batch accuracy and ensure inter-batch standardization. The coefficient of variation for the cadmium measurements was ≤5.0%. External quality control was maintained via participation in the National Quality Control Program conducted by the government of Taiwan.

According to the following BCL value tertiles, all enrolled patients were divided into 3 groups for statistical analysis: low (1^st^ tertile) BCL (<0.19 μg/L), middle (2^nd^ tertile) BCL (0.19–0.39 μg/L), and high (3^rd^ tertile) BCL (>0.39 μg/L).

### Laboratory, nutritional, and inflammatory parameters

All laboratory values, including blood cell counts, biochemical data, normalized protein nitrogen appearance (nPNA), peritoneal transport characteristics, residual renal creatinine clearance (Ccr), peritoneal Ccr, standard total weekly Ccr (normalized to body surface area), residual renal Kt/V urea, peritoneal Kt/V urea, and total Kt/V urea, were measured by automated and standardized methods. All blood samples were collected in the morning after at least 12 h of fasting. Serum albumin, creatinine, cholesterol, triglyceride, hemoglobin, serum ferritin, and transferrin saturation levels were assayed and recorded. Serum calcium levels were corrected for serum albumin levels using the following formula: Corrected calcium (mg/dL) = serum calcium (mg/dL) + 0.8 × [4.0 - serum albumin (g/dL)]. Serum albumin levels were used as a marker of nutrition status. Serum ferritin and high-sensitivity C-reactive protein (Hs-CRP) levels were used as markers of inflammation. Serum Hs-CRP concentrations were measured via immunonephelometry (Nanopia CRP; Daiichi, Inc., Tokyo, Japan), with the lowest detection limit of *<*0.15 mg/L. All other markers were measured using an automatic analyzer according to standard laboratory methods.

### Definition of malnutrition and inflammation

This study defined malnutrition as serum albumin levels of *<*3.6 g/dL (*<*36 g/L), consistent with previous literature [10,13]. Since the lack of any definite Hs-CRP cutoff level indicated an inflammatory state in CPD patients, the presence of inflammation was defined as an Hs-CRP level of *>*3 mg/L, which correlates with increased cardiovascular risk in the general population [18].

### Statistical analysis

Unless otherwise stated, continuous variables are expressed as the mean ± standard deviation (SD), and categorical variables are expressed as the number or percentage for each item. Comparisons between the 3 study groups were analyzed via trend tests. Logarithmic conversion was performed for non-normally distributed variables, including BCLs (log BCLs), serum ferritin (log ferritin), Hs-CRP (log Hs-CRP), residual renal Ccr (log renal Ccr), and intact parathyroid hormone (iPTH) level (log iPTH).

To identify factors associated with log BCLs, we applied a simple linear regression analysis for all variables. All potential variables (*P* < 0.05) assessed with simple linear regression were entered into multiple linear regression models with backward stepwise procedures. To evaluate the variables related to malnutrition and inflammation, we applied univariate and multivariate logistic regression analysis to assess the odds ratio (OR) and 95% confidence interval (CI) of these variables. All potential variables (*P* < 0.05) assessed by univariate logistic regression analysis were entered into multivariate logistic regression models with forward stepwise procedures. The level of significance was set at *P* < 0.05. Data were analyzed using the Statistical Package for the Social Sciences (SPSS) version 12.0 for Windows 95 (SPSS Inc., Chicago, IL, USA).

## Results

### Study population characteristics

A total of 301 CPD patients (112 men and 189 women) with a mean PD duration of 3.6 ± 2.9 years were enrolled for analysis. The mean patient age was 49.5 ± 13.5 years (range, 19–88 years). Based on the tertile of increased BCLs, the subjects were stratified into 3 groups: the low BCL (<0.19 μg/L) group, containing 101 subjects; the middle BCL (0.19 - 0.39 μg/L) group, containing 102 subjects, and the high BCL (>0.39 μg/L) group, containing 98 subjects. Table 1 lists the demographic and clinical characteristics, including age, gender, body mass index (weight/height^2^), presence of DM and CVD, and the biochemical and hematological data of the 3 groups. The mean serum albumin and blood hemoglobin levels were 3.9 ± 0.5 g/dL (range, 1.1–5.0 g/dL) and 8.5 ± 1.4 g/dL (range, 4.2–13.6 g/dL), respectively. The median serum Hs-CRP level was 5.25 mg/L (range, 0.20–127.61 mg/L). Compared with the patients in the low and middle BCL groups, those in the high BCL group were older and had a higher prevalence of DM but lower serum albumin, creatinine, and phosphate levels. No differences existed between the 3 groups in terms of sex, body mass index, smoking status, presence of hypertension, or history of CVD. The PD duration and peritoneal membrane solute transport type were also not different. Furthermore, the 3 groups did not differ in terms of erythropoietin dose, total Kt/V, residual renal weekly Ccr, standard weekly total Ccr, nPNA, hemoglobin, alanine aminotransferase (ALT), transferrin saturation, ferritin, serum corrected-calcium, iPTH, cholesterol, triglyceride, and Hs-CRP levels, as well as the presence of viral hepatitis B antigen and viral hepatitis C antibody (data not shown).

**Table 1:** **Baseline characteristics according to blood cadmium level tertile in CPD patients (*n*** **= 301)**

**Characteristics**	**Low BCL group (<0.19 μg/L)**	**Middle BCL group (0.19 - 0.39 μg/L)**	**High BCL group (>0.39 μg/L)**	***P***
	**(*n*** **= 101)**	**(*n*** **= 102)**	**(*n*** **= 98)**	
*Demographics*				
Age (years)	45.3 ± 13.1	50.1 ± 13.1	53.2 ± 13.3	<0.001
Female sex	58 (57.4)	66 (64.7)	65 (66.3)	0.192
Body mass index (kg/m^2^)	22.7 ± 3.6	22.7 ± 4.2	22.7 ± 3.6	0.799
Smoking (Yes)	23 (22.8)	26 (25.5)	19 (19.4)	0.567
*Comorbidity*				
Diabetes mellitus (Yes)	7 (6.9)	13 (12.7)	17 (16.7)	0.023
Hypertension (Yes)	90 (89.1)	88(86.3)	84 (85.7)	0.468
Previous cardiovascular diseases (Yes)	14 (13.9)	13 (12.7)	11 (11.2)	0.183
*Dialysis-Related Data*				
Peritoneal dialysis duration (years)	3.8 ± 2.9	3.4 ± 3.1	3.8 ± 2.9	0.609
High and high average Cr D/P, 4 h (Yes)	44 (43.6)	58(56.9)	49 (50.0)	0.658
Erythropoietin (U/kg/week)	45.9 ± 32.5	44.5 ± 31.5	45.6 ± 31.0	0.810
Total Kt/V	2.17 ± 0.33	2.18 ± 0.42	2.16 ± 0.37	0.478
Residual renal Ccr (L/week/1.73 m^2^)*	2.51 (0.0, 71.9)	2.84 (0.0, 68.1)	2.99 (0.0, 56.4)	0.452
Standard total Ccr (L/week/1.73 m^2^)	63.8 ± 14.5	61.9 ± 13.4	62.9 ± 14.1	0.365
nPNA (g/kg/day)	1.09 ± 0.28	1.07 ± 0.26	1.05 ± 0.26	0.303
*Biochemical Data*				
Hemoglobin (g/dL)	8.44 ± 1.56	8.53 ± 1.36	8.66 ± 1.32	0.250
Albumin (g/dL)	3.96 ± 0.46	3.85 ± 0.45	3.78 ± 0.50	0.006
ALT (IU/L)	16.5 ± 16.2	19.0 ± 12.2	19.7 ± 16.0	0.131
Creatinine (mg/dL)	12.8 ± 3.0	12.2 ± 2.9	11.6 ± 3.1	0.005
Transferrin saturation (%)	27.8 ± 13.6	27.0 ± 14.2	28.2 ± 12.3	0.835
Ferritin (μg/L)*	207.0 (6.9, 1930.0)	170.0 (5.9, 1497.0)	196.0 (7.0, 1909.0)	0.950
Corrected calcium (mg/dL)	10.0 ± 0.9	10.1 ± 0.8	10.2 ± 1.0	0.081
Phosphate (mg/dL)	5.5 ± 1.5	5.1 ± 1.3	5.0 ± 1.3	0.010
Intact parathyroid hormone (pg/mL)*	107.0 (5.0,998.0)	83.2 (5.0, 647.0)	81.0 (5.0, 1187.0)	0.165
Hs-CRP (mg/L)*	7.09 (0.20,124.45)	3.89 (0.26, 127.61)	3.52 (0.20, 59.8)	0.863
*Cardiovascular Risks*				
Total cholesterol (mg/dL)	200.4 ± 44.5	209.6 ± 51.1	208.3 ± 41.9	0.223
Triglyceride (mg/dL)	214.1 ± 158.3	200.3 ± 131.7	190.2 ± 130.5	0.233

Note: *P* < 0.05 represents significant trends between the groups. *Non-normally distributed data are presented as median (minimum, maximum). Patients with hypertension were either taking antihypertensive drugs regularly or their blood pressure was >140/90 mmHg at least twice. Cardiovascular diseases included cerebral vascular disease, coronary arterial disease, congestive heart failure, and peripheral vascular disease. ALT, alanine aminotransferase; BCL, blood cadmium level; CPD, chronic peritoneal dialysis; Ccr, clearance of creatinine; Cr, creatinine; Cr D/P, dialysate-to-plasma ratio of creatinine; Hs-CRP, high-sensitivity C-reactive protein; nPNA, normalized protein nitrogen appearance.

### Dialysate cadmium concentrations, peritoneal cadmium clearance, and BCLs of healthy volunteers and CPD patients

The median cadmium level was 0.09 μg/L (range, 0.01–0.19 μg/L) in all dialysate samples (*n* = 13), which was compatible with the American Association for Advancement of Medical Instrumentation Standards (cadmium: <10 μg/L). After a 4-h abdominal retention in the 13 CPD patients, the median cadmium level of the dialysates was 0.10 μg/L (range, 0.03–0.27 μg/L). In all study subjects, the mean BCL was 0.53 μg/L and the median BCL was 0.27 μg/L (range, 0.02–9.98 μg/L). In healthy volunteers, the mean BCL was 0.27 μg/L and the median BCL was 0.15 (range, 0.02–1.03)μg/L.

### Determinants of BCLs in CPD patients

As shown in Table 2, simple linear regression analysis revealed that log BCLs were positively associated with age, PD duration, DM, and ALT potentially, but negatively associated with albumin, creatinine, and phosphate levels potentially.

**Table 2:** **Determinants of blood cadmium levels (log BCLs) in CPD patients (*n*** **= 301)**

	**Simple linear regression**		**Backward stepwise multiple regression analysis**	
**Variables**	**Coefficient ± SE**	***P***	**Beta Coefficient ± SE**	***P***
Age (years)	0.009 ± 0.002	<0.001	0.006 ± 0.002	0.001
Peritoneal dialysis duration (years)	0.017 ± 0.008	0.040		
Diabetes mellitus (Yes = 1)	0.171 ± 0.077	0.028		
Albumin (g/dL)	-0.142 ± 0.053	0.008	-0.167 ± 0.034	0.002
ALT (IU/L)	0.004 ± 0.002	0.030	0.003 ± 0.002	0.046
Creatinine (mg/dL)	-0.027 ± 0.008	0.001		
Phosphate (mg/dL)	-0.052 ± 0.019	0.006		

ALT, alanine aminotransferase; CPD, chronic peritoneal dialysis; iPTH, intact parathyroid hormone; Log BCL, logarithmic transformation of the blood cadmium level; SE, standard error.

After adjusting for these potential variables, stepwise backward multiple linear regression analysis revealed that only age and ALT levels were positively and significantly associated with log BCLs and only albumin levels (beta coefficient ± standard error [SE] = -0.167 ± 0.034, *P* = 0.002) were negatively and significantly associated with log BCLs.

### Determinants of serum albumin levels in CPD patients

As shown in Table 3, simple linear regression analysis revealed that serum albumin levels were positively associated with hemoglobin, cholesterol, triglyceride, creatinine, and phosphate levels potentially, but negatively associated with age, DM, high and high average dialysate-to-plasma ratio of creatinine (Cr D/P) at 4 h, log BCLs, and log Hs-CRP levels potentially.

**Table 3:** **Determinants of serum albumin levels in CPD patients (*n *** **=301)**

	**Simple linear regression**		**Backward stepwise multiple regression analysis**	
**Variables**	**Coefficient ± SE**	***P***	**Beta Coefficient ± SE**	***P***
Age (years)	-0.011 ± 0.002	<0.001		
Diabetes mellitus (Yes = 1)	-0.302 ± 0.081	<0.001		
High and high average Cr D/P, 4 h (Yes = 1)	-0.264 ± 0.053	<0.001	-0.202 ± 0.064	0.002
Hemoglobin (g/dL)	0.046 ± 0.017	0.008	0.050 ± 0.021	0.020
Cholesterol (mg/dL)	0.002 ± 0.001	0.001	0.002 ± 0.001	0.044
Triglyceride (mg/dL)	0.001 ± 0.000	0.001	0.001 ± 0.000	0.001
Creatinine (mg/dL)	0.055 ± 0.008	<0.001	0.047 ± 0.012	<0.001
Phosphate (mg/dL)	0.097 ± 0.019	<0.001		
Log BCL (μg/L)	-0.277 ± 0.059	<0.001	-0.185 ± 0.074	0.013
Log Hs-CRP (mg/L)	-0.113 ± 0.056	0.045	-0.095 ± 0.052	0.043

CPD, chronic peritoneal dialysis; Cr D/P, dialysate-to-plasma ratio of creatinine; Log BCL, logarithmic transformation of the blood cadmium level; Log Hs-CRP, logarithmic transformation of the high-sensitivity C-reactive protein level.

After adjusting for these potential variables, stepwise backward multiple linear regression analysis revealed that only hemoglobin, cholesterol, triglyceride, and creatinine levels were positively and significantly associated with serum albumin levels and only high and high average Cr D/P at 4 h, log Hs-CRP, and log BCLs (beta coefficient ± SE = -0.185 ± 0.074, *P* = 0.013) were negatively and significantly associated with serum albumin levels.

Specifically, in logistic regression analysis, the log BCLs (OR = 2.64, 95% CI: 1.07–6.48, *P* = 0.035) were significantly associated with malnutrition (serum albumin levels of <3.6 g/dL) in the forward stepwise multivariate logistic regression analysis.

### Determinants of log Hs-CRP levels in CPD patients

Simple linear regression analysis demonstrated that age, standard total weekly Ccr, serum albumin, triglyceride, creatinine, and transferrin saturation levels were potentially associated with log Hs-CRP levels (*P* < 0.05). After adjusting for these potential variables, backward stepwise multiple linear regression analysis revealed that age (beta coefficient ± SE = 0.010 ± 0.004, *P* = 0.005) and standard total weekly Ccr (beta coefficient ± SE = 0.006 ± 0.003, *P* = 0.050) were positively and significantly associated with log Hs-CRP levels, but transferrin saturation levels (beta coefficient ± SE = -0.009 ± 0.003, *P* = 0.005) were negatively and significantly associated with log Hs-CRP levels.

## Discussion

The analytical results presented in this study demonstrated an association between BCLs and nutritional status and malnutrition in CPD patients. Following adjustment for potential variables, BCLs were negatively correlated with serum albumin levels and malnutrition in these patients. Overall, each 10-fold increase in BCL was associated with a decrease of 0.17 g/dL in the serum albumin levels of CPD patients. Moreover, each 10-fold increase in BCL was associated with a 2.64-fold increase in the probability of malnutrition development (serum albumin levels <3.6 g/dL) in these subjects. Since all subjects with cadmium poisoning, occupational exposure to cadmium, and living in cadmium-contaminated areas or near the cadmium-using factories were excluded, this study is the first to demonstrate that environmental cadmium exposure is significantly associated with nutritional status and malnutrition in CPD patients.

Reviewing the published articles, there were similar studies performed in MHD population. A recent study in Japan [19] revealed that cadmium accumulation in hair correlated with malnutrition in 60 MHD patients. Furthermore, our previous study [10] also presented that environmental cadmium exposure is significantly associated with malnutrition in 954 MHD patients. Since malnutrition may cause protein-energy wasting and is associated with increased risk of mortality in ESRD patients [14,15], it seems reasonable to imply that environmental cadmium exposure may influence the nutritional status and survival of CPD patients. Therefore, regular BCL measurements may be required to assess protein-energy wasting and mortality in CPD patients. However, the definite underlying pathogenesis requires further investigation.

This study also first demonstrated that CPD patients with high BCLs were older and had lower serum albumin and creatinine levels than those with lower BCLs. In addition to the negative association between log BCLs and the nutritional marker of serum albumin levels, stepwise backward multiple linear regression analysis revealed positive associations between log BCLs and age and ALT levels in CPD patients. Regardless of the route of exposure, cadmium is progressively retained in the organism and accumulates throughout life. The cadmium body burden, negligible at birth, increases continuously during the lifespan until the approximate age of 60 to 70 years [4]. Hence, it seems reasonable to suggest that age is positively associated with cadmium in CPD patients. Cadmium accumulates in the liver and even more so in the kidneys, which can contain up to 50% of the total cadmium body burden in individuals with low levels of environmental exposure [4]. There are reports indicating that cadmium-induced toxicity results from the generation of free radicals [20,21]. Additionally, an increase of free radical formation may also be observed in patients with CPD [22]. Free radicals lead to lipid peroxidation, which might induce organ damage and structural change [23]. Moreover, the liver is a critical target organ of acute or chronic exposure to cadmium [24]. Hence, it seems reasonable to consider that ALT levels may be positively associated with BCLs in CPD patients. Factors that impact the liver function may result in the release of cadmium from the liver, which may increase the BCL [17]. However, further study is needed to clarify the mechanism.

Although the literature [25,26] indicates that low iron body stores or calcium status may enhance cadmium absorption and increase BCLs in mice and humans, the current study demonstrated that patients in the high BCL group did not exhibit significantly lower corrected levels of calcium, ferritin, and transferrin saturation, compared with those in the other 2 groups. Hence, BCLs were not correlated with serum corrected calcium and iron levels in the study subjects. These results may be explained by the fact that dialysis patients regularly receive iron supplements for anemia to maintain erythropoietin effects and obtain calcium from the calcium-containing phosphate binders and dialysate. However, further studies are needed to clarify these observations.

In general population, cigarette smoking is significantly correlated with cadmium levels [2]. However, the high BCL group did contain a significantly higher percentage of smokers compared with those in the other 2 groups in this study. Unlike the general population with normal kidneys, accumulation of cadmium in CPD patients may be caused by the nearly complete loss of renal function in the ESRD population and the difficulty of cadmium removal under current dialysis settings [7,9,27]. This may be the reason for which the smoking status did not differ significantly between the 3 study groups.

On reviewing the published articles, the different levels of serum CRP between patients with CPD and those with MHD are controversial. In this investigation, the median Hs-CRP level (5.25 mg/L; range, 0.20–127.61 mg/L) of CPD patients was higher than that (2.88 mg/L; range, 0.20–73.21 mg/L) of MHD patients in our previous study [10]. This result was similar to a previous study [28], which presented that the mean CRP level (12.8 ± 9.7 mg/dL) was higher in CPD patients than that (8 ± 4.5 mg/dL) in MHD patients. The reasons underlying the high Hs-CRP levels of CPD patients may be that (1) the inflammatory markers in these subjects can be enhanced both by the peritoneal irritation during PD and the decreased removal of the cytokines due to reduced residual renal function [29], and (2) some MHD patients (197/954 = 20.6%) received hemodiafiltration with ultrapure dialysate, which may reduce the Hs-CRP elevation in dialysis sessions [30]. However, other studies demonstrated that patients with MHD had higher CRP or Hs-CRP levels than patients with CPD [31,32]. Additionally, other studies showed that the levels of CRP or Hs-CRP were similar between patients with MHD and those with CPD [33,34]. Therefore, further studies are needed to clarify this issue.

In this investigation, the BCL was not associated with inflammation in CPD patients. However, in our previous study [10] of MHD patients, the BCL was significantly associated with inflammation in the dialysis population. The reason for which BCL is associated with inflammation in MHD patients but not in CPD patients may be the lower median BCL (0.27 μg/L; range, 0.02–9.98) of CPD patients relative to that (0.39 μg/L; range, 0.02–16.45, *n* = 954) of MHD patients. Moreover, only 9.4% of non-smoking CPD patients had BCLs of >1 μg/L, while 17.8% of non-smoking MHD patients had BCLs of >1 μg/L, a level indicating abnormal concentrations in humans as reported in a previous study [2]. Since PD and HD cannot effectively remove cadmium from the body, the reasons underlying why CPD patients have a lower BCL than MHD patients remain unknown. There are several potential explanations for this observation: (1) the younger age (49.3 ± 13.5 years) of CPD patients versus MHD patients (56.1 ± 13.5 years), suggesting lower BCLs in CPD patients since BCL increases continuously throughout life; (2) lower serum albumin levels in CPD patients (3.9 ± 0.5 g/dL) than in MHD patients (4.1 ± 0.4 g/dL), which indicates a relatively poor intake in CPD patients compared to MHD patients, suggesting lower dietary cadmium ingestion in CPD patients; (3) HD duration of 6.7 ± 0.2 years versus PD duration of 3.6 ± 2.9 years, as a longer duration of dialysis may cause increased cadmium accumulation in the body; (4) the residual daily urine amount ≧ 100 mL is 63% (190/301) in CPD patients versus 20.9% (199/954) in MHD patients, indicating elevated cadmium excretion in the urine of CPD patients; and (5) the hemoglobin level (8.5 ± 1.4 g/dL) in CPD patients is lower than that (10.9 ± 1.6 g/dL) of MHD patients, and since most cadmium binds to hemoglobin in red blood cells, CPD patients with lower hemoglobin levels may have lower BCLs compared to MHD patients. Moreover, since no similar reports in CPD patients are noted, we only can compare the data with those of the general population and MHD patients. The geometric mean BCL was 0.53 μg/L in our study subjects, which is higher than that (0.44 μg/L) of the general population in America [35] and (0.27 μg/L) of the general population in the study area, but similar to that (0.51 μg/L) of the general population in Europe [36]. Considering normal hemoglobin in the general population, the BCL of CPD patients, similar to the previous report in MHD patients [10], was higher than those of general population. A previous report indicated that the exposure to relatively low environmental levels of cadmium appears to be a determining factor in the development of ESRD [37]. Moreover, in patients undergoing chronic dialysis, cadmium elimination is very difficult because of their very low residual renal function and the inefficient cadmium removal by the current dialysis settings. All these may be contributing factors to the higher BCL found in patients with CPD compared to the general population in the study area. However, further study is needed to clarify these observations.

Cadmium is a ubiquitous environmental pollutant in industrialized countries [4]. The cadmium levels of in air due to contaminated fumes or dust vary greatly between different industries, such as smelters, pigment plants, and battery factories [2]. Compared with the general population, workers in the non-ferrous industries are exposed to higher levels of cadmium, mainly through inhalation of contaminated substances with other toxins [4]. Moreover, the application of phosphate fertilizers may lead to the contamination of soils and then increase the absorption of cadmium by crops and vegetables, grown for human consumption [2].

Diet is the main source of environmental cadmium exposure in the non-smoking population in most countries [38]. Cadmium is present in all foods virtually in varied concentrations, depending on the kind of food and the cadmium levels in the environment. High cadmium concentrations are observed in mollusks and crustaceans such as oysters and other bivalve mollusks, cephalopods, and crabs [38]. High cadmium levels are also present in offal products of visceral organs, especially from livers and kidneys of older animals, as well as in oil seeds, cocoa beans, and certain mushrooms [4,38]. Plant products generally contain higher cadmium levels than animal products like meat, egg, milk, and fish [38]. Moreover, among vegetables, green leafy vegetables, cereals, potatoes, and roots, such as carrots, contain higher cadmium concentrations than other vegetables [38]. Tobacco smoking is another important source of cadmium exposure. One cigarette may contain approximately 1–2 μg of cadmium [38]. The results of the current study indicate that avoiding the intake of cadmium-rich foods and tobacco smoking is critical for the survival of long-term dialysis patients. Additionally, the mean BCL of 63 healthy volunteers in our study area was 0.27 μg/L, which was lower than the mean BCLs in the general population of America (0.44 μg/L) and Europe (0.51 μg/L). The reason for the lower mean BCL found in the study area compared to that found in America and Europe is uncertain. However, the industrial emissions, the application of fertilizer, sewage sludge, cigarette smoking, and dietary habits may influence the mean BCL of the study population. Further study is needed to clarify this observation.

In ESRD patients, there are many causes of osteomalacia, including vitamin D deficiency, acidosis, uremic toxins, and aluminum accumulation in bone [39]. However, the serum concentrations of iPTH may be at any level and only bone histology can establish the diagnosis of osteomalacia secondary to dialysis with certainty [40]. Additionally, iPTH levels may be clinically influenced by several other factors, such as the administration of phosphate-binders, use of calcium-containing dialysates, and supplementation of vitamin D3. For these reasons, although cadmium may induce osteomalacia in a clinical setting, a relationship between cadmium and iPTH levels could not be observed in this study. However, further study is needed to clarify the underlying mechanism.

Cadmium is a cumulative toxic metal with a long elimination half-life [10]. In acute exposure, inhalation of cadmium oxide fumes may induce inhalation fever or chemical pneumonitis [41]*.* In chronic exposure, cadmium poisoning may cause renal tubulopathy, induce osteomalacia, and diffuse osteoporosis [41]. Furthermore, the International Agency for Research on Cancer (IARC) has classified cadmium as a carcinogen in humans [41].

Due to the cross-section nature of this study, the causal relationship between BCLs and malnutrition was not identified. Further investigation is required to clarify whether cessation of possible environmental exposure, including smoking and ingestion of cadmium-rich foods, may improve the nutritional status and reduce the malnutrition rate of CPD patients. Moreover, the small number of patients in each group, the higher mean age, and absence of an adequate control group are also limitations of this study. Based on the results of previous studies [4,38,42], blood cadmium has a very long half-life (approximately 7–16 years) in humans [42] and BCLs are well correlated with urinary cadmium levels [4,38]. Therefore, BCLs and urinary cadmium levels may reflect the total cadmium burden. The long half-life of cadmium is due to the influence of cadmium body accumulation on the BCL. Thus, after long-term low-level exposure, BCL measurement offers a useful means to estimate the total body burden of cadmium, as it is closely related to the total body burden of cadmium in the general population [4,35,38]. Since CPD patients with high-level cadmium exposure were excluded from the current study, the BCLs of the patients in our study, who only had long-term low-level cadmium exposure, may represent their total body cadmium burden. Therefore, BCL was a good index of the total body burden of cadmium in the CPD patients in this study. Hence, the results of our study indicate that chronic environmental low-level cadmium exposure may play a role in the increased malnutrition of CPD patients.

## Conclusions

This is the first study to demonstrate that BCLs are related to age, serum albumin levels, and ALT levels in CPD patients. Moreover, BCLs were negatively associated with nutritional status and related to high probability of malnutrition development in CPD patients. Additionally, the World Health Organization has classified cadmium as a human carcinogen [2], and cancer is a common cause of mortality in ESRD population [43]. These results indicate that reducing environmental cadmium exposure, particularly by avoiding smoking and the intake of cadmium-rich foods, may be critical for the improvement of nutritional status and survival of these patients. However, further investigations are needed to clarify and confirm these observations.

## Abbreviations

ALT: Alanine aminotransferase; BCLs: Blood cadmium levels; Ccr: Creatinine clearance; CGMH: Chang gung memorial hospital; CI: Confidence interval; CPD: Chronic peritoneal dialysis; Cr D/P: Dialysate-to-plasma ratio of creatinine; CVD: Cardiovascular disease; DM: Diabetes mellitus; ESRD: End-stage renal disease; Hs-CRP: High-sensitivity C-reactive protein; iPTH: Intact parathyroid hormone; MHD: Maintenance hemodialysis; nPNA: Normalized protein nitrogen appearance; OR: Odds ratio; PD: Peritoneal dialysis; SD: Standard deviation; SE: Standard error; SPSS: Statistical package for the social sciences.

## Competing interest

All authors declared that they have no competing interest.

## Authors’ contributions

CWH and JLL formed the study concept, interpreted the results, and drafted the manuscript. DTLT and WHH collected and analyzed the data. KHC analyzed the data and revised the manuscript. THY provided the statistical expertise and comments on the manuscript drafts. All authors read and approved the final manuscript.

## Pre-publication history

The pre-publication history for this paper can be accessed here:

http://www.biomedcentral.com/1471-2369/15/17/prepub
